# Colliding ideals – an interview study of how intervention researchers address adherence and adaptations in replication studies

**DOI:** 10.1186/s12874-018-0496-8

**Published:** 2018-05-08

**Authors:** Ulrica von Thiele Schwarz, Ulrika Förberg, Knut Sundell, Henna Hasson

**Affiliations:** 1 Medical Management Centre, Department of Learning, Informatics, Management, & Ethics, Karolinska Institutet, Stockholm, Sweden; 2 School of Health, Care and Social Welfare, Mälardalen University, Västerås, Sweden; 3 Children’s and Women’s Health Theme, Karolinska University Hospital, and Department of Women and Child Health, Karolinska Institutet, Stockholm, Sweden; 4 Center for Epidemiology and Community Medicine, Stockholm County Council, Stockholm, Sweden

**Keywords:** Adherence, Adaptations, Fidelity, Intervention research, Modifications, Efficacy, Effectiveness, Sweden, Systematic reviews, Research synthesis

## Abstract

**Background:**

For an intervention to be considered evidence-based, findings need to be replicated. When this is done in new contexts (e.g., a new country), adaptations may be needed. Yet, we know little about how researchers approach this. This study aims to explore how researchers reason about adaptations and adherence when conducting replication studies, describe what adaptations they make and how these are reported in scientific journals.

**Methods:**

This was an interview study conducted in 2014 with principal investigators of Swedish replication studies reporting adaptations to an intervention from another country. Studies (*n* = 36) were identified through a database of 139 Swedish psychosocial and psychological intervention studies. Twenty of the 21 principal investigators agreed to participate in semi-structured telephone interviews, covering 33 interventions. Manifest content analysis was used to identify types of adaptations, and qualitative content analysis was used to explore reasoning and reporting of adaptations and adherence.

**Results:**

The most common adaptation was adding components and modifying the content to the target population and setting. When reasoning about adaptations and adherence, the researchers were influenced by four main factors: whether their implicit aim was to replicate or improve an intervention; the nature of evidence outlying the intervention such as manuals, theories and core components; the nature of the context, including approaches to cultural adaptations and constraints in delivering the intervention; and the needs of clients and professionals. Reporting of adaptations in scientific journals involved a conflict between transparency and practical concerns such as word count.

**Conclusions:**

Researchers responsible for replicating interventions in a new country face colliding ideals when trying to protect the internal validity of the study while considering adaptations to ensure that the intervention fits into the context. Implicit assumptions about the role of replication seemed to influence how this conflict was resolved. Some emphasised direct replications as central in the knowledge accumulation process (stressing adherence). Others assumed that interventions generally need to be improved, giving room for adaptations and reflecting an incremental approach to knowledge accumulation. This has implications for design and reporting of intervention studies as well as for how findings across studies are synthesised.

## Background

Replicating studies is a vital part of the scientific process of accumulating knowledge [1]. It has been recommended that at least two rigorous trials must have shown an intervention to be efficacious, in order to avoid building recommendations on findings due chance or specific to a time, place or person [2]. From this follows that users of research evidence are encouraged to base their decisions on systematic synthesis of research, e.g. systematic reviews and meta-analyses, rather than individual studies [3, 4]. This puts replication at the centre of the research-to-practice pathway.

Replications can be direct (an exact copy of the study) or conceptual, which involves testing the intervention with different methods or, more commonly, in different contexts, thus investigating the generalisability of the findings [5, 6]. Direct replication requires adherence, whereas conceptual replications imply some type of adaptations to the original intervention, target population or setting. In a global world, conceptual replications may involve testing the intervention in a new country, where differences in terms of care systems, norms, regulations and cultures are to be expected [7]. In this, researchers need to consider whether it is possible to follow the original intervention protocol or if adaptations are needed in order to make the intervention work in the new context. Adaptations may be particularly relevant for interventions that consist of several components that interact with each other as well as with factors related to the implementation and context where they are set (i.e. complex interventions) [8]. Previous research into the adherence and adaptation dilemma has focussed on how and why professionals adapt evidence-based methods, but little is known about how researchers approach this issue.

According to the principle of programme uniqueness [9], interventions are being developed and evaluated under circumstances that are different from where they will be used (e.g., in funding, homogeneity of patients and training of staff). It can be argued that this makes direct replication of interventions impossible; instead, re-testing of interventions will involve changes in various aspects of the intervention or context [9].

This, in combination with the fact that interventions are seldom sufficiently described to determine the degree of adherence and adaptations [10–14], imposes challenges for knowledge accumulation, because what seems like the same intervention may in fact be fundamentally different versions of it. The more variation in how the interventions are composed (and the less that is known about it), the greater the challenge of determining how to categorise interventions in systematic reviews [8]. Unknown variation between interventions that on the paper are the same may result in erroneous conclusions when results from individual studies are synthesised. In addition, information about how variations in intervention components, such as when they are adapted to fit different contexts, is lost [8]. Such information is central for decision makers and professionals who need to sort out what methods work in their context. Thus, the way in which adaptation and adherence are approached and reported in replication studies has implications for what is being reproduced and, in the end, what interventions patients receive and the likelihood that these will benefit them. However, there is a lack of studies on how adaptation and adherence are approached in replication studies.

Empirical findings support that interventions might require adaptations when used in new contexts, such as a new country, in order to obtain positive outcomes. For example, a recent meta-analysis evaluating the effects of evidence-based youth psychotherapies showed that the interventions were no longer effective when they were applied, without any adaptations, in other countries [15]. The authors concluded that evidence-based methods may not generalise well beyond their culture of origin and that adaptations may be needed. Another meta-analysis comparing interventions that were applied in a new country with or without adaptations showed that although non-adapted versions were effective, the adapted versions were more effective [16]. This is in line with meta-analysis and reviews from the field of culturally adapted interventions covering psychotherapy, substance abuse and family interventions, showing that culturally adapted interventions are at least as effective as non-adapted ones, while generally being superior in recruiting and retaining minority groups (see, for example, [17–19]). A recent systematic review of evidence-based psychotherapies showed similar results, indicating that adapted interventions generally were effective, albeit few of them tested against the original protocol [20].

In line with these findings, several researchers argue that adaptations are necessary at all steps of the research-to-practice pathway [21, 22]. Yet, while there is emerging knowledge about what type of adaptations professionals make and why (e.g., [12, 23, 24]), less is known about adaptations when researchers evaluate interventions in new contexts. Thus, this study aims to describe adaptations that researchers make when conducting replication studies and to explore how they reason about adaptations and adherence and report adaptations in scientific journals. To our knowledge, this is the first study on researchers’ views on adherence and adaptations in replication studies.

## Method

### Design and setting

This was an interview study conducted in 2014 with principal investigators of Swedish replication studies reporting adaptions to an intervention transported from another country (mostly the USA). The studies were identified from a database consisting of randomised and non-randomised intervention studies with a pre-post- or pre-follow-up design conducted in Sweden (*N* = 139) and published in scientific journals between 1990 and 2012. The interventions concerned psychological or social (i.e. behavioural health) interventions and targeted individuals with physical, psychological or social problems (both prevention and rehabilitation).

Two persons independently reviewed all of the articles in the database to identify replication studies of interventions from another country that reported adaptations defined according to Stirman et al.’s framework, that is, including adaptations in content, procedure, dosage, setting, format or target population [12]. Inter-coder agreement for the identification of adapted replication studies versus other studies was 93%. Disagreements were solved through discussion. This process resulted in 36 studies reporting adaptations. Thus, all studies that were identified as 1) replications and 2) reporting adaptations, were included.

All 21 principal investigators for the 36 studies (some researchers were principal investigators for several studies) were invited to participate. Of these, 20 principal investigators responsible for 33 studies accepted. Seven were responsible for more than one study. The 33 studies represented six research areas: psychology (16 studies), psychiatry, substance abuse and public health (4 from each), medicine (3), social work (2), and criminology (1). The principal investigators were from 9 Swedish universities. The studies were published in 23 different peer-review journals between 1994 and 2013. Sixty-one percent of the studies targeted indicated populations (i.e., tertiary prevention), 33% targeted universal prevention (i.e., primary prevention), and the rest, 6%, targeted selective prevention (i.e., secondary prevention). The studies targeted 14 distinct areas, with substance abuse as the most prevalent (33%), followed by antisocial behavior and eating disorders, (both 12%). The remaining interventions targeted areas such as depression, PTSD, heart problems and pain. Approximately half of the studies (53%) were trials under “real world” clinical settings (i.e., effectiveness trials), and the other half (47%) were conducted under ideal conditions (i.e., efficacy trials) [25]. Of the studies, 76% had a randomised design, and in most cases (70%), subjects in the control condition received another active treatment. The target group consisted primarily of adults (70%).

### Data collection and analysis

The interviews were semi-structured with questions concerning views on adherence and adaptations in general and in relation to the specific studies. Stirman et al.’s (2013) framework was used to probe for adaptations. The interview guide was pilot-tested with other intervention researchers, resulting in minor changes. All interviews were conducted by a clinical psychologist over telephone and lasted between 19 min to 33 min. The interviews were recorded and transcribed verbatim.

Manifest content analysis was used to identify types of adaptations, and qualitative content analysis with an inductive approach [26] was used to explore reasoning and reporting of adaptations and adherence. The second author performed the condensation of the text into meaning units and subcategories, and all authors took part in condensing the subcategories into main categories.

## Results

### Types of adaptations

The types of adaptations that were made to the interventions are described in Table 1. The decision was typically made by the researcher (67%) and involved changes in the target population (42%) or setting (36%). The nature of adaptations included adding components (45%), followed by removing (33%) and tailoring (24%) components.

**Table 1:** Types of adaptations made to interventions (*n* = 33) categorized according to Stirman et al. [12]

Adaptations	Frequency^a^
Decision maker	
Individual practitioner (professionals)	4
Team /multiple professionals	10
Administrator or supervisor	0
Researcher	22
Purveyor or intervention developer	2
Coalition of stakeholders	5
Contextual adaptation	
Intervention format	8
Setting	12
Type of personnel	6
Target population	14
Nature of adaptation	
Tailoring/tweaking/refining	8
Adding elements	15
Removing elements	11
Shortening/condensing	5
Lengthening/extending	2
Substituting elements	1
Re-ordering elements	0
Integrating another approach into the intervention	0
Integrating the intervention into another approach	0
Departing from the intervention	0
Loosening structure	0
Repeating elements	0

^a^More than one alternative may be applicable

### Reasoning about adaptations

**Fig. 1:**
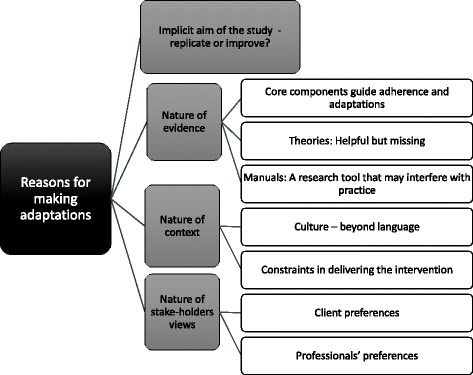
Researchers’ reasoning about adaptation and adherence summarised in four main categories and eight subcategories

The researchers voiced a wide range of reasons both for striving for adherence and for doing adaptations, resulting in four main categories: reasons related to 1) the aim of the inquiry, 2) the nature of the evidence, 3) the nature of the context and 4) the nature of stakeholders’ needs. These, and the eight subcategories, are summarised in Fig. 1 and described below.

#### Aim of the inquiry: Replication or intervention improvement?

The respondents’ attitudes towards adherence and adaptations differed depending on if their implicit aim was to replicate previous findings or to improve the intervention. For replication, adherence was described as a way to ensure that the same intervention was evaluated as in the original study, thereby contributing to establishing a stable knowledge base. This was needed before any adaptations could be considered. In contrast, researchers emphasising the goal of improving the intervention viewed adaptations as an innate part of making sure that interventions evolve over time.

*“Well, from a methodological perspective, I believe that one should adhere if one has decided to do a replication. Now, for our study, I guess the main aim was not to replicate but rather to search for methods that had worked and also had certain shortcomings, and then we attempted to address those shortcomings.” (Interview 2).*

#### Nature of evidence

In this category, three subcategories emerged.

##### Manuals – a research tool that may interfere with practice

Intervention manuals could be both helpful and troublesome in relation to adherence and adaptations. On the one hand, manuals facilitated adherence, and, with adherence to a manual, one knew what one was evaluating.

*“From my point of view, a manual is a way to ensure that you to a larger extent do the things you assume are the effective things in the method. Manuals, when they describe what you should do, then they are sort of means to help people.” (Interview 9).*

On the other hand, manuals could also be too rigid, making one preoccupied by following them at the expense of other things, such as client needs. Furthermore, the researchers noted that researchers and clinicians may perceive manuals differently. While adherence to a manual may be valuable from an evaluation perspective (i.e., the researchers), manuals may restrict professionals, thus making them lose the flexibility needed to adapt the intervention in response to the client or contextual needs. In the end, they may even risk implementing something that is not in the client’s best interest. This realisation also led some respondents to invite clinicians to make some adaptations to the manual.

*“It is both good and not so good to stay very faithful to a manual. What is good about it is that you do not miss or forget anything. What is bad about being too faithful to a manual is that you stand the risk of implementing something that you really should have avoided.” (Interview 20).*

##### Theories: helpful but missing

While few respondents mentioned the theoretical underpinnings of interventions in relation to adaptations, those who did found theories essential for knowing what to adapt and not and that it was problematic that they were often overlooked.

*“… [this thing] about fidelity or not fidelity, and about adaptations versus no adaptations, cannot really be discussed without bringing up the theoretical foundations, what would you call it, the constitutional law for the method.” (Interview 15).*

##### Core component guide adherence and adaptation

Core components or similar concepts (active ingredients, essential components etc.) were frequently referred to as essential for managing adaptations and adherence. Adaptations were acceptable as long as they did not interfere with the core components, as that would compromise the result and risk turning the intervention into something else. Knowing about and adhering to core components determined whether a study is a replication, a development or reinvention or a conceptually new intervention.

*“It is like a stew. If you make a beef stew, then it is the beef and the tomato sauce that are the main ingredients. If these ingredients are kept in the stew, then you can call it a beef stew. You can then adjust the seasoning and add one vegetable or the other…but if you all of a sudden add fish instead, even with the tomato sauce, sorry, then it is not a beef stew anymore, it is a fish stew.” (Interview 12).*

Nevertheless, it was noted that the core components were seldom described in the original articles and seldom empirically tested. Instead, the respondents reported using theory and other empirical studies to identify core components.

*“We know so little about what actually works in different methods. Most often, we have tested the whole packages to see if they work or not. Dismantling studies, they are scarce.” (Interview 15).*

#### Nature of context

##### Cultural adaptations – beyond language

The most immediate response to questions about adaptations concerned translations to another language. In addition, the need to adapt interventions to the Swedish context so that it makes sense for the target group was often mentioned. Not all intervention components were perceived as suitable, which motivated adaptations. The decision was conscious but made on gut feelings or preconceptions, rather than data or an explicit theory. Such cultural adaptations often involved changing examples but could also involve removing or tweaking content changes that the researchers did not feel would fit in the Swedish context.

*“Many of these exercises were very American in the original version. It was a bit more toned-down in the Swedish version.” (Interview 18).*

##### Constraints in delivering the intervention

Adaptations were also justified by constraints that the new context imposed on how the intervention could be delivered. Sometimes, adaptations were perceived as a prerequisite for delivering the intervention at all. The constraints could be at different levels and involve different types of contextual modifications (i.e., in format, setting, personal and population). On the national level, constraints were, for example, differences in legislation, how social and health services are organised and the variation in professional roles and training between countries. Regional or local factors also imposed constraints, such as when the intervention was delivered in a more rural area as compared to the original study.

*“Well, we made many adaptations to relate to the Swedish law and context, one could say. We were in a small town and included all patients that we met, including both easier and more difficult cases, whereas the method originally addresses more difficult cases.” (Interview 8).*

It was also noted that some interventions are so interdependent of the context that it becomes impossible to disentangle them and that these interventions simply cannot be transported. That is, the intervention may be specific to a certain context.

*“Sometimes these programmes aren’t worth adapting, because they don’t work in another context. [xxx]. I have looked at people who have been involved in many programmes, developed in the US. They’ve tried them here, and the programmes haven’t worked, and it’s not because they’re being adapted in the wrong way, but it’s because the society is different.” (Interview 22).*

#### Nature of stakeholders’ needs

##### Client preferences

The respondents acknowledged that they had felt compelled to adapt the intervention in order to meet clients’ needs and preferences, either for the whole client population or for individual clients. If interventions were not adapted, the interventions may only be applicable to a limited group of clients that may not be representative for the whole population.

*“I am very conscientious about the target group’s needs and wishes. You have to be responsive to what the target group needs and wants, because our reoccurring problem with these type of interventions is that we only reach a small group, and the group that we reach may not be representative for everyone.” (Interview 10).*

On the individual level, the fact that clients differ was acknowledged. Doing exactly the same for all clients was sometimes described as impossible or not appropriate.

*“Well, you try to apply the methods equally the best you can. However, it is impossible to do the exact same thing for different individuals. You have to adapt to the different clients as well.” (Interview 13).*

Many reported leaving room for some degree of flexibility for professionals to decide on adaptations to patient needs. Yet, few mentioned providing any direction about the degree of variability that the professional had, or any guidance, towards what this variability may entail in terms of concrete adaptations, maybe because these adaptations were assumed to be small.

##### Professionals’ preferences

The respondents also reflected on the fact that, for interventions that are delivered by human beings, variation between those who deliver the intervention is expected.

*“Someone says: we should deliver this service, this intervention. The fact that it may deviate from the original plan, well, I guess that’s the way it is, to be a human being.” (Interview 18).*

### Reporting on adaptations in scientific journals

In reporting adaptations in publications, transparency was the key word. This was raised repeatedly. It was argued that deviations from protocols are normal but require transparency so that the intervention can be replicated and stimulate new research.

*“I think it’s important to describe adaptations that might influence the outcome. I think it’s extremely important to do that. You have to—I mean, in theory, there shouldn’t be anything that stops you from doing that, no. Otherwise, you are sort of withholding something that could influence the outcome, which is not appropriate.” (Interview 22).*

Despite the emphasis on reporting adaptations, this was not always done. Particularly, those perceived as minor or those perceived to be completed to facilitate implementation were not always reported. Minor adaptations were not viewed as important enough to merit the cost of a tedious reporting that decreases readability. Descriptions of cultural adaptations to a small country such as Sweden were also considered a risk, potentially making the study irrelevant for people from other countries.

*“If we are too local in our way of describing…then we are perceived as strange northerners, and doing ourselves a disservice. Therefore, if I am going to be quite honest, which I am with you now, it is almost the case of toning down that we even make adaptations.” (Interview 20).*

Also, factors in the publication process mattered for the degree to which adaptations were described. The most common factor was word counts, forcing the researchers to choose what and how they could report adaptations. Furthermore, journal guidelines and editors perceived as indifferent to reporting of adaptations could also impact reporting. The researchers also acknowledged that it was sometimes difficult to know if one should describe an intervention as a direct replication (adopted without adaptations), an adapted, or a new intervention. This was related to the difficulties in determining the extent of adaptations that can be done without an intervention being fundamentally different. Copyright to intervention was also a factor – you may be inclined to refer to a method as a new one to avoid running into legal problems.

*“There are many who call it their own methods just with a few adjustments, which has to do with copyright and things like that.” (Interview 1).*

## Discussion

This study addresses how researchers who replicate interventions in another country reason about adherence and adaptations. The findings suggest that evaluating interventions in new contexts introduces a conflict between adhering to the original intervention protocol and making adaptations to make the intervention fit the context. The resolution of this conflict was influenced by the researchers’ implicit aim of the inquiry, the nature of the evidence underlying the intervention, the context to which the intervention was transferred and the target stakeholders’ (professionals and clients) views. Reporting was also conflictive, as the wish to be transparent clashed with practical constraints such as word count. How the conflict between adherence and adaptations is approached has several implications for intervention research, as well as for how findings are synthesised across studies (e.g., in systematic reviews and meta-analyses). This is discussed below.

Despite the fact that all included studies were replications in that they involved evaluating a previously tested intervention in a new context, the findings indicate that the researchers’ implicit aims with their studies could differ. Two types of implicit aims emerged: 1) to replicate an intervention by repeating a previous study as closely as possible, albeit in a new context (i.e. a conceptual replication), or 2) to incrementally improve an intervention, making sure learning from previous experiences was harvested when testing in a new context. These two aims seem to reflect two divergent approaches to knowledge accumulation. Those stating that the aim of the study was to replicate a previous intervention emphasised that successful reproduction of the original study is a necessary step to ensure that findings are trustworthy, before being spread and implemented in practice. With this approach to knowledge accumulation, adherence is a core feature. The approach aligns with the way the research-to-practice pathway is currently set up, with the focus on internal validity before external and the gradual process of establishing efficaciousness through a series of distinct steps, from development of interventions and feasibility studies to direct and conceptual replication of studies, first under optimal conditions (efficacy-studies) and then normal conditions (effectiveness) [2, 27].

The second approach, incrementally improving the intervention, has other implications for the accumulation of knowledge and the research-to-practice pathway. Rather than aiming to establish a stable knowledge base through repetition, the researchers described how each study was set up to refine the knowledge base, which is in line with Roger’s notion of reinvention [28]. This kind of replication study is, thus, neither a direct replication (test of the exact same intervention in the same type of context but by another research group), nor a typical conceptual replication, where previously tested interventions are re-tested in different populations or contexts [5, 6]. Instead, this strategy for accumulation of knowledge can be described as *incremental*, based on the emphasis on continuously improving interventions and ultimately outcome over testing the boundaries or generalisability of the original intervention. In this, incremental accumulation does not follow the distinct steps of first developing, then testing interventions, but rather a continuous development-testing loop.

The strategy for knowledge accumulation – accumulation through replication (direct or conceptual) or incremental accumulation – has implications for how interventions are designed, analysed and reported. Whereas investigating the intervention as an entity may be feasible for replications without adaptations, we suggest that adaptations and the subsequent incremental approach to knowledge accumulation require more focus on understanding the impact of different intervention components and intervention interaction with context. This would change the research question from if an intervention as a whole is efficacious, to what intervention components and combinations of components and contexts result in a certain outcome, as well as what works for whom, when and why.

Increased understanding of intervention components and interaction between component and contextual factors can be done either by alternative ways of analysing data, without changing the design of the trial, or by alternative designs of the trial. Alternative ways of analysing the data include dismantling studies, component analysis, mediation and moderation analysis, realist evaluation, and Bayesian statistics [29–32]. Examples of alternative designs are adapted (flexible) designs, factorial designs, randomized micro-trials and hybrid designs as well as tailored interventions and implementations [33–37]. These suggestions are in line with calls for designs that make intervention studies more responsive to societal changes [38]. It is also in line with a recent review of adaptations to evidence-based psychotherapies, which concluded that alternative designs are needed to illuminate the impact of specific adaptations, and gives specific design recommendations for how this can be achieved [20]. Such studies, may, for example, include testing adapted versus non-adapted versions in the same trial [7]. None of the researchers in this study reflected on this possibility. Rather they seemed to treat the adaptation-adherence dilemma more as a discourse, not an empirical question to test.

Incremental accumulation of knowledge may also call for complementary strategies to research synthesis. To reconcile the need to both summarize evidence and retain details about interventions and the context in which they are used, a number of alternative strategies have been developed. For example, by integrating program logic models with systematic reviews, core components, change mechanisms and contextual influences can be explicated [39, 40]. Specific varieties of such approaches are realist synthesis and qualitative comparative analysis which aims to illuminate what works for whom when and under what circumstances [41, 42]. Other approaches that may be useful when knowledge accumulation is incremental are meta-regression, which allows analysis of moderators across studies, network meta-analysis and mixed treatment comparisons [43]. These are methods that allow three or more interventions (or versions of interventions) to be compared instead of only contrasting effects between intervention and control groups [44, 45].

In addition to the findings showing that the researchers’ implicit aim of the inquiry influenced how they reasoned about adherence and adaptations, the researchers were also influenced by the nature of the evidence underlying the intervention, the context to which the intervention was transferred and the target stakeholders’ (professionals and clients) views. All three themes can be described to be dealing with the need to create a practical, philosophical or cultural fit between the intervention and the context where it was set. Adaptations can be viewed as the tool to create this fit. This expands previous findings reporting that creating fit between intervention and context is an important reason for why practitioners make adaptations [23, 24]. It also shows that the fit concept, which has previously been studied extensively in organizational research, including fit between the organisational environment (e.g., work processes) and people [46], and between interventions and organisation and its members [47], is also applicable in the context of intervention research. As adaptations may be a way to achieve fit, they may be integral in making interventions work in new settings. Overall, the fact that researchers may need to consider adaptations regardless of their strategy for knowledge accumulation indicated that the adherence and adaptation issue is central for understanding how interventions are conducted not only in clinical practice but also in replication studies. It also underlines the importance of describing interventions, contexts and adaptations in greater detail, as encouraged in recent reporting guidelines [48].

The respondents did not describe any efforts to control, monitor or support how adaptations were made by the professionals involved in the intervention studies. This was despite the fact that they often anticipated that the professionals were going to make adaptations. This is in contrast to how the researchers, for example, used manuals to support adherence. The combination of using manuals to support adherence whilst neglecting to control, monitor or support adaptations may increase the risk of adaptations being done ad hoc or in a way that is conceptually inconsistent with the intervention, something that is common in clinical practice (e.g [23]). This calls for intervention researchers to focus more on monitoring adaptations, both planned and unplanned, so that these can be described and analysed. There is a need to support professionals in conducting adaptations so that they are made proactively and in line with the logic of the intervention. There are several frameworks that can be used in this regard, providing systematic, theoretically guided approaches to adaptations (e.g., [49–52]). There is also a need to support professionals in monitoring adaptations as the intervention unfolds, providing them with a feedback system that makes it possible to manage adaptations in the light of client progress [53–55].

Reporting adaptations in scientific journals evoked a conflict between the norm of transparency and the practical reality. The respondents described how in theory, transparency was non-negotiable and all adaptations made to interventions should be reported. However, in practice, minor adaptations were not mentioned; the word limits and fear of obscuring the story made overly detailed descriptions impossible. As most adaptations were perceived as minor, many were left out. However, this is risky, particularly when core components are not known. Even though the adaptations may be perceived as minor by the researcher, they may in fact be critical to intervention success or for the professionals aiming to use the method in their practice [56]. Overall, the reporting of adherence and adaptations in peer-reviewed articles did not seem to do justice to the deliberations that the researchers vocalised.

### Methodological considerations

This study is exploratory in its nature. Several limitations might have biased the results and our conclusions. One is that the participants were identified based on information available in articles; only principal investigators of studies reporting adaptations were invited to participate. This might have biased the sample towards researchers who were more aware of the need to report adaptations or who were published in journals that encouraged this type of reporting. It is also possible that their answers were influenced by social desirability, and as always with interviews, it was their subjective experience that was in focus; it is possible that others involved in the different studies would have provided contrasting perspectives.

In addition, some of the studies were conducted more than 20 years ago, raising the issue of memory bias. Some respondents pointed out that adaptation was not on the agenda at the time of their study. Thus, it is possible that the reporting of adaptations may be more frequent in later studies, or that current knowledge about adaptations might have distorted the original motives for adaptation. Furthermore, this study primarily deals with psychological and social interventions (behavioural health interventions) in health and social care, and all studies were conducted in Sweden. Nevertheless, the sample did cover a broad range of different interventions with different target groups and thus did not focus on only one specific method.

## Conclusions

This study adds to the limited knowledge about how and why researchers make adaptations and how they reason about adherence and adaptations when conducting replication studies. The findings show that adherence and adaptations are related to implicit assumptions about the role of the trial and suggest it matters if the goal is 1) to test an intervention in a new context to confirm or disconfirm those findings, or 2) to expand or limit the application of the intervention, making sure learning from previous experiences is harvested by improving the intervention. The latter goes beyond what is usually involved in so-called conceptual replications because not only the context where the study is set varies, but also the intervention. As the goal is improvement rather than reproduction, we call this strategy for accumulation of knowledge “incremental accumulation”. We suggest that direct and conceptual replications and incremental accumulation require different approaches to adaptation and adherence; adherence being central to direct replications and adaptations to incremental accumulation. As incremental replications may involve variation in intervention components as well as variation in, and interaction with the context, methodologies and designs that allow this variation to be studied, not controlled, is warranted.

To be able to accumulate findings from incremental replications in systematic reviews, there is a need for alternative approaches also at this stage of the research-to-practice pathway. By increasing the awareness of the implicit aims underlying replication of interventions, a more systematic consideration of how one best accumulates knowledge in systematic reviews can be achieved. In addition, regardless of the type of replication, the findings suggest that interventions often need to be adapted to fit the context of application, for practical or cultural reasons. Thus, adaptations need to be monitored and reported, not only adherence. Lastly, the participants acknowledged that professionals and clients may often make adaptations. This points to a greater need for the research community to provide structured support for adaptations, not only for adherence.
